# The Formation of a Novel Intergeneric Hybrid Fish Derived from *Megalobrama amblycephala* (♀) *× Culter dabryi* (♂)

**DOI:** 10.3390/ani15223302

**Published:** 2025-11-15

**Authors:** Zhifeng Zhou, Xinge Ouyang, Chang Wu, Siyu Fan, Faxian Yu, Liran Zhang, Xinxin Yu, Zhong Tang, Lang Qin, Yi Zhou, Shengnan Li, Ming Wen, Yuequn Wang, Min Tao, Shaojun Liu

**Affiliations:** 1 Engineering Research Center of Polyploid Fish Reproduction and Breeding of the State Education Ministry, College of Life Sciences, Hunan Normal University, Changsha 410081, China; 2 Yuelushan Laboratory, Changsha 410128, China

**Keywords:** distant hybridization, *Megalobrama amblycephala*, *Culter dabryi*, genetic inheritance, growth and reproductive, nutritional composition

## Abstract

Distant hybridization in fish serves to integrate the genetic material from two distinct, distantly related species. In this study, we successfully produced a new hybrid fish (BG) through the intergeneric hybridization of blunt snout bream (*Megalobrama amblycephala*, BSB, 2n = 48, ♀) and green tip culter (*Culter dabryi*, GTC, 2n = 48, ♂). The objective of this research was to characterize the genetic, morphological, reproductive, and nutritional features of the hybrid compared with its parents. The DNA content and chromosomal number analysis revealed that BG was a diploid hybrid fish with 48 chromosomes. Integrated analysis of 5S rDNA, mitochondrial DNA, and *Sox9* gene sequences revealed that BG predominantly inherited its genetic traits from BSB. Notably, certain gene fragments (376 bp segment of 5S rDNA class II and 718 bp segment of BG *Sox9*-II) originated from the paternal GTC, demonstrating biparental genomic integration. The growth performance analysis revealed that BG exhibited enhanced growth, achieving a body weight significantly greater than that of BSB and 1.81-fold that of GTC (both *p* < 0.05). The reproductive analysis indicated that BG possessed bisexual fertility, with testicular histology revealing accelerated spermatogenesis relative to BSB. Additionally, the nutritional analysis of BG muscle revealed elevated levels of crude protein (18.13%) and umami amino acids (5.45%) compared to those in its parents. BG showed higher growth, bisexual fertility, and improved muscle nutritional composition. This hybrid represents a promising resource for cyprinid breeding and aquaculture diversification.

## Simple Summary

In this study, we successfully developed a novel hybrid fish (BG) based on distant hybridization between blunt snout bream (BSB, ♀) and green tip culter (GTC, ♂). Genetic characterization confirmed that BG was diploid (2n = 48), exhibiting predominantly maternal (BSB) genetic inheritance with the incorporation of specific paternal (GTC) genomic fragments. BG demonstrated a significantly higher body weight, reaching approximately 1.81-fold that of the GTC. Additionally, it possessed bisexual fertility and accelerated spermatogenesis compared to BSB. Nutritional analyses demonstrated that BG contained a higher crude protein content, increased levels of umami amino acids, and greater concentrations of beneficial polyunsaturated fatty acids than its parents. Collectively, these results indicate that BG combines superior growth performance, reproductive characteristics, and nutritional qualities, establishing its significant potential as an advantageous germplasm resource for aquaculture breeding programs.

## 1. Introduction

Distant hybridization, often referred to as “macro-hybrid”, is a technique extensively employed in aquaculture breeding to facilitate crossing between species from distinct genetic backgrounds [1]. This approach enables the production of hybrid offspring that combine subgenomes from both parents, often leading to heterosis in traits such as growth rate, disease resistance, and reproductive performance [2]. Distant hybridization has been utilized for germplasm improvement in the *Megalobrama* species, with the aim of developing novel varieties with enhanced aquaculture potential.

Blunt snout bream (*Megalobrama amblycephala*, BSB) is highly favored for its fast growth and tender meat. However, its sustainable cultivation is currently threatened by severe germplasm degradation, manifested as reduced growth performance, weakened disease resistance, and lower breeding efficiency. Furthermore, the lack of improved varieties and advanced breeding technologies has further constrained the expansion of its large-scale aquaculture [3]. Green tip culter (*Culter dabryi*, GTC) is rich in nutrients, has a delicate meat quality, and a short sexual maturation cycle, which contributes to shortening the hybridization breeding period [4]. Nevertheless, population declines and genetic deterioration have been driven by habitat alteration and overexploitation. To address these challenges, this study conducted intergeneric hybridization between BSB and GTC. The aim was to produce hybrid offspring with superior aquaculture traits, thereby providing an effective approach for genetic improvement and sustainable resource utilization of both species.

However, intergeneric hybridization between these two species has not yet been characterized genetically or phenotypically. To precisely characterize the genetic background and potential value of the hybrid, we employed multiple molecular markers. The 5S ribosomal DNA (5S rDNA) is a polygene family of tandem repeat units containing a highly conserved 120 bp coding sequence (5S rRNA) and a variable non-transcriptional spacer (NTS) [5]. The 5S rRNA demonstrates remarkable species sequence conservation, while the NTS displays significant species specificity, making 5S rDNA an important molecular marker for investigating species evolution and phylogenetic relationships [6]. The *Sox* gene family, as another important molecular marker, possesses a highly conserved high mobility group (HMG) box domain, which facilitates specific DNA binding and participates in transcriptional regulation [7]. Mitochondrial DNA (mtDNA), as a simple and highly stable extranuclear genetic material, plays a pivotal biological role in eukaryotes [8]. The mtDNA is characterized by its rapid evolutionary rate, strict maternal inheritance, and no genetic recombination. These unique properties make it particularly advantageous for research in species identification, population genetics, and genetic diversity [9,10]. Collectively, these markers enable a comprehensive assessment of the hybrid’s genetic composition, inheritance patterns, and evolutionary relationships.

Muscle nutritional quality is a critical breeding target. Fish products are a high-quality animal food source, rich in protein and unsaturated fatty acids with high biological value. Freshwater fish is rich in high-quality protein and its essential amino acid content is very close to human requirements, highlighting its importance in human nutrition [11]. Fat is an important component of the human diet. Fish is rich in essential fatty acids (EFAs), especially long-chain n-3 polyunsaturated fatty acids (LC n-3 PUFA). The human body cannot synthesize these components, meaning they must be obtained from the diet [12]. Distant hybridization is considered a promising strategy for the potential improvement of nutritional components in fish. Studies have indicated that it can play a positive role in enhancing overall nutritional value [13]. In this study, we therefore compared the proximate composition, amino acid, and fatty acid content in BG and its parents to determine whether hybridization also improved nutritional attributes.

This study successfully produced a novel hybrid (BG) via distant hybridization between BSB and GTC. It was designed to systematically characterize the hybrid by examining their formation and ploidy, elucidating genetic inheritance patterns, assessing fertility levels, and comparing growth performance and muscle nutritional traits. This research provides a valuable germplasm resource for future studies in fish genetic breeding.

## 2. Materials and Methods

### 2.1. Acquisition and Hybridization Preparation of Experimental Fish

BSB and GTC in this study were cultured at the Hunan Fish Genetics and Breeding Center. We used ecological breeding cages combined with artificial dry fertilization technique to facilitate fish spawning. During the breeding season of 2022, when the water temperature was above 25 °C, 10 males and 10 females from BSB and 10 males and 10 females from GTC were selected as parental subjects for the hybridization experiment. The hybrid combinations in this study included female BSB × male BSB, female BSB × male GTC, and female GTC × male GTC. As the objective of this work was to evaluate the potential for germplasm improvement of BSB via hybridization, the reciprocal cross (female GTC × male BSB) was omitted. Utilizing BSB as the maternal parent enables a direct assessment of hybrid performance within a BSB-oriented breeding framework. Ten female BSB and ten female GTC were injected with a mixture of the hormones containing HCG (1000 and 2000 IU/kg, respectively), LHRH-A2 (10 μg/kg), and DOM (2 mg/kg). Conversely, ten male BSB and ten male GTC received a mixture of the hormones without DOM. For these males, the doses of HCG and LHRH-A2 were reduced to half of those administered to their female counterparts (500 and 1000 IU/kg HCG, and 5 μg/kg LHRH-A2, respectively). The fertilized eggs developed in a tank with a water temperature of 25~27 °C, maintaining water flow and providing sufficient dissolved oxygen. We recorded the whole embryonic development process of BG from fertilized eggs to hatching using a stereomicroscope equipped with digital imaging. The fertilization rate, hatching rate and hatching period were also calculated, and all of the above experiments were the subject of more than three repetitions.

### 2.2. Measurement of Morphological Traits

Twenty BSB, twenty GTC, and twenty BG individuals, all 6 months old with good growth statuses, were randomly selected. The methods for measuring morphological characteristics were based on previous studies [14]. Six countable growth traits, namely lateral scale number, upper lateral scale number, lower lateral scale number, dorsal fin number, ventral fin number, and anal fin number, were visually assessed. Seven measurable growth traits including whole length (WL), body length (BL), body height (BH), head length (HL), head height (HH), caudal peduncle length (CPL), and caudal peduncle width (CPH) were measured using a ruler and caliper. These seven measurable growth traits were transformed into six standardized traits: the ratios of the whole length to body length (WL/BL), body length to body height (BL/BH), body length to head length (BL/HL), head length to head height (HL/HH), body height to head height (BH/HH), and caudal peduncle length to caudal peduncle height (CPL/CPH). All of the experiments described above were conducted in triplicate.

### 2.3. Measurement of DNA Content

To determine the ploidy level, tail venous blood samples were collected from 20 BSB, 20 GTC and 20 BG at 6 months of age. We used flow cytometry (manufactured by Partec, Münster, Germany) to determine the DNA content of the venous red blood cells of BSB, GTC, and BG. Blood samples were prepared according to the method described by Wu et al. [15]. The DNA content of BSB was used as a control and the DNA content of each of the three samples was measured under the same conditions. All of the experiments described above were conducted in triplicate.

### 2.4. Preparation of Chromosome Spreads and Karyotype Analysis

To further determine the chromosomal karyotype of BG, we collected the kidney tissues of 20 BSB, 20 GTC, and 20 BG. Metaphase chromosome slides were prepared according to previously described methods, followed by staining with Giemsa stain [16,17]. We randomly observed chromosome morphology and counted 100 metaphase spreads of each fish with a microscope under an enlarged 100 × (Leica DM2500 LED, Leica Microsystems, Wetzlar, Germany) oil lens. At the same time, the well-spread metaphase chromosomes were photographed and used for karyotype analysis. All of the experiments described above were conducted in triplicate. Classification was performed according to the ratio of the long-arm to the short-arm of the chromosome, as described in the previous literature [18].

### 2.5. Examination of 5S rDNA and Sox Genes

Twenty BSB, twenty GTC, and twenty BG red blood cell samples were selected from the collected tail vein blood. The total genomic DNA was extracted using the Universal Genomic DNA Extraction Kit (TaKaRa, Dalian, China). The quality and concentration of DNA were determined by means of agarose gel electrophoresis and ultraviolet spectrophotometry. Specific primers for 5S rDNA and *Sox* genes, as reported by Qin and Chen, were used for amplification. The primer sequences were as follows: 5S-F (5′-GCTATGCCCGATCTCGTCTGA-3′) and 5S-R (5′-CAGGTTGGTATGGCCGTAAGC-3′) for 5S rDNA, and *Sox*-F (5′-TGAAGCGACCCATGAA(C/T)G-3′) and *Sox*-R (5′-AGGTCG(A/G)TACTT(A/G)TA(A/G)T-3′) for the *Sox* genes. The amplification and purification procedures followed those described in the previous literature [19,20]. We recovered and purified the 5S rDNA and *Sox* gene bands from the three fish species. Following this, we selected 20 monoclonal clones from each purified band for sequencing. DNA sequencing was performed, and sequence homology analysis was conducted using BioEdit (v7.0.9.0) and DNAMAN (v9.0).

### 2.6. Mitochondrial DNA Sequence Analysis

Peripheral blood cells from twenty GTC and twenty BG specimens were collected, and total genomic DNA was extracted using the Universal Genomic DNA Extraction Kit (TaKaRa, Dalian, China). The primers for amplifying the full-length mitochondrial DNA of GTC and BG were designed based on the complete mitochondrial DNA sequence of BSB (NCBI accession: MF522177.1). The design was performed using Primer Premier 5.0 software, with reference to experimental methods from previous literature [21]. All primers used for the mitochondrial genome sequences of GTC and BG are listed in Table S1. The fragments’ amplification, recovery, and purification were performed as described in Section 2.5. The amplified mitochondrial sequences were analyzed using the NCBI ORF Finder (https://www.ncbi.nlm.nih.gov/orffinder; accessed on 15 March 2024), BioEdit, and EditSeq software for identifying open reading frames (ORFs), determining gene size and initiation sites, and performing a statistical analysis of relative codon proportions, respectively. The sequence homology analysis was conducted using DNAMAN. The full-length mitochondrial BSB was obtained from the GenBank database under accession number MF522177.1. Referring to the methods described in the previous literature, the mitochondrial annotation and map construction of BSB, GTC, and BG were performed using the online software MitoAnnotator (https://mitofish.aori.u-tokyo.ac.jp/annotation/input; accessed on 15 March 2024) [22].

### 2.7. Measurement of Growth Performance

In mid-June 2024, following egg hatching and progression into the post-larval stage characterized by horizontal swimming capability, fry of BSB, GTC, and BG were systematically transferred into identical ceramic tanks. A completely randomized design was employed to assign each of the three experimental groups (BSB, GTC, and BG) to three replicate tanks, resulting in a total of nine tanks. Each tank had a volume of approximately 420 L. The stocking density was standardized at 100 individuals per tank, resulting in an initial total of 300 fish per group. To ensure uniform environmental conditions across all experimental tanks, pond water was replenished daily at a constant flow rate through individual valves connected to a shared pipeline system. Supplementary of water quality logs and food composition are provided in the Supplementary Explanation S4. After a rearing period of 180 days, all surviving fish in each tank were counted to assess survival rate. Subsequently, 20 fish were randomly selected from the combined survivors of the three replicate tanks per group for body weight measurement, with an accuracy of 0.01 g, to enable the precise assessment of growth performance.

### 2.8. Gonad Microstructure Analysis

To determine the reproductive performance of BG, we used paraffin sections of gonadal tissue for fertility analysis. Twenty healthy specimens each of BSB, GTC, and BG at 6 months and 15 months of age, respectively, were anesthetized, euthanized, and dissected. Gonadal tissues were dissected and fixed in Bonn’s fixative. The tissues were then processed through dehydration in a graded ethanol series, clearing in xylene, and paraffin embedding. Subsequently, sections were cut and stained with hematoxylin and eosin (H&E) for microscopic examination [17,23]. All of the experiments described above were conducted in triplicate. We evaluated their stages of gonadal development on the basis of the criteria previously established for fishes of the *Cyprinidae* family [24].

### 2.9. Preliminary Analysis of Nutritional Components in Muscle of BG and Its Parents

For each group (BSB, GTC, and BG), nine individuals (*n* = 9) with comparable body weight and standard length were randomly selected and anesthetized. Dorsal muscle tissues from both sides of each fish were excised, immediately frozen in liquid nitrogen, and stored at −80 °C. Each biological sample was analyzed in triplicate for nutrient composition. Protein content was measured using the Kjeldahl method (GB 5009.5-2016), while fat content was measured using the Soxhlet extraction method (GB 5009.6-2016) (For detailed information on the standards, please refer to Supplementary Explanation S6.). Amino and fatty acids were analyzed using an automatic amino acid analyzer (Hitachi; LA8080; OpenLAB CDS 2.3) and a gas chromatograph (Agilent; 7890A; OpenLAB CDS 2.5) according to national standards and instrument instructions. All measurements were performed in triplicate, and the results were expressed as g/100 g.

The Amino Acid Score (AAS), Chemical Score (CS), and Essential Amino Acid Index (EAAI) serve as key indicators for assessing the nutritional quality of proteins. The amino acid content was calculated by converting the amino acid content in the muscle of the three fish species into the mg number of amino acids per 1 g N. Then, the amino acid content was compared with the amino acid score standard recommended by FAO/WHO and the amino acid pattern of egg protein. The AAS, CS, and EAAI calculation formulas and unit conversions were provided in the Supplementary Explanation S5 [25,26,27].

### 2.10. Heterosis Calculation and Statistical Analysis

In this study, mid-parent heterosis (MPH) and superior-parent heterosis (SPH) were calculated to quantify the level of heterosis for each trait. Specifically, MPH was calculated as the percentage deviation of the F_1_ mean from the mid-parent value (MP, calculated as (P_1_ + P_2_)/2) using the formula MPH (%) = [(F_1_ − MP)/MP] × 100. Similarly, SPH was determined as the percentage by which the F_1_ mean exceeds the mean of the higher-performing parent (HP) for each trait, given by SPH (%) = [(F_1_ − HP)/HP] × 100. These calculations were performed for each cross combination based on the mean phenotypic values obtained from replicated experiments.

Statistical analyses were performed using IBM SPSS Statistics 23.0 and GraphPad Prism 8.0.1. Data were presented as mean ± standard deviation (SD). All comparisons in this study were between two groups and were therefore conducted using the *t*-test (all *t*-tests were two-tailed, with the significance level set at α = 0.05.). Statistical significance was denoted by asterisks (*): * *p* < 0.05, ** *p* < 0.01, and *** *p* < 0.001. The effect size was estimated using Hedges’ g, with values of 0.2~0.5, 0.5~0.8, and ≥0.8 conventionally representing small, medium, and large effects, respectively.

## 3. Results

### 3.1. The Formation and Embryonic Development of BG Hybrid Fish

**Figure 1:**
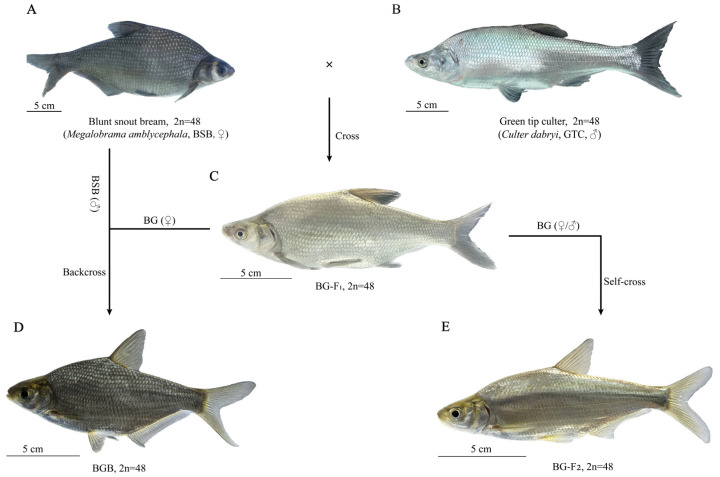
The hybridization process and appearance of the BG hybrid fish. (**A**) The appearance of blunt snout bream (BSB). (**B**) The appearance of green tip culter (GTC). (**C**) The appearance of BG. (**D**) The appearance of BGB. (**E**) The appearance of BG-F_2_.

The hybridization process of the BG hybrids is shown in Figure 1. Figure S1 depicts the embryonic development of BG. At a stable water temperature of 26~27 °C, the time required for BG to emerge from the membrane was 31 h and 44 min. The specific developmental time course is shown in Table S2. Under identical temperature conditions, BG’s hatching time occurred approximately 2 h earlier than in BSB (♀) × BSB (♂). The fertilization rates, hatching rates, and hatching period of BSB, GTC, and BG hybrids are presented in Table S3.

### 3.2. Morphological Characteristics

The appearances of BSB (Figure 1A), GTC (Figure 1B), and BG (Figure 1C) are shown in Figure 1. In terms of morphology, the body height of BG was similar to that of BSB, while the head was smoother and more similar to that of GTC. The comparison of the measurable morphological traits is presented in Table 1.

BG exhibited traits from both parents, with the number of lateral scales and abdominal fins falling between those of BSB and GTC. Other countable traits tended to be biased towards the female parent, BSB. The comparison of the countable morphological traits is provided in Table 2.

Except for the WL/BL ratio, the quantitative trait ratios of BG were between those of BSB and GTC. Compared with BSB, BG showed significant differences in the BH/HH and CPL/CPH ratios (*p* < 0.05), with a highly significant difference in the BL/BH ratio (*p* < 0.001). Compared with GTC, BG exhibits significant differences in the BL/BH and BH/HH ratios (*p* < 0.01) and a significant difference in the BL/HL ratio (*p* < 0.05). There were no significant differences in the other ratios between BG and its parent species (*p* > 0.05).

### 3.3. DNA Content of BSB, GTC, and BG

**Figure 2:**
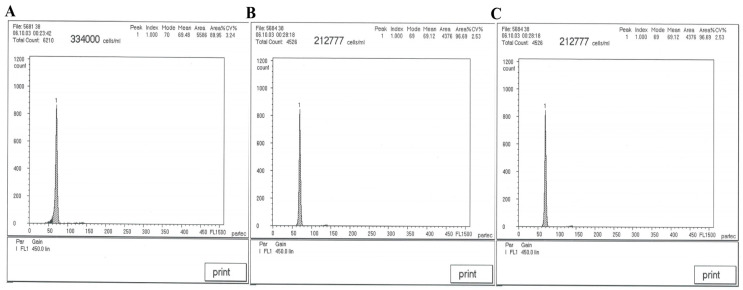
The cytometric histograms of BSB, GTC, and BG. (**A**) The average DNA content of BSB (peak 1: 69.48). (**B**) The average DNA content of GTC (peak 1: 69.12). (**C**) The average DNA content of BG (peak 1: 68.72). Note: n = 20 per group.

The DNA contents and cytometric histograms of BSB, GTC, and BG are presented in Table 3 and Figure 2. According to the data in the analysis table, there was no significant difference in the average DNA content between BG, BSB, and GTC (*p* > 0.05), with the ratio between them being close to 1:1.

### 3.4. Chromosome Number and Karyotype Analysis

**Figure 3:**
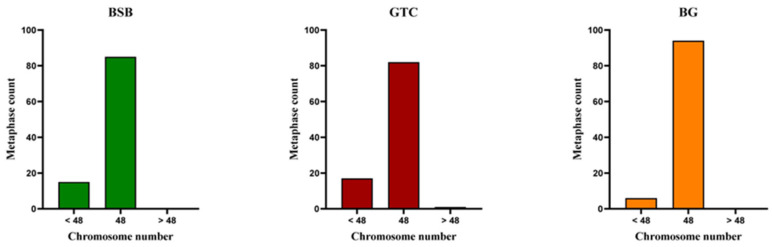
Distribution of chromosome numbers in 100 metaphases of BSB, GTC, and the BG hybrid. Note: n = 100 per group.

**Figure 4:**
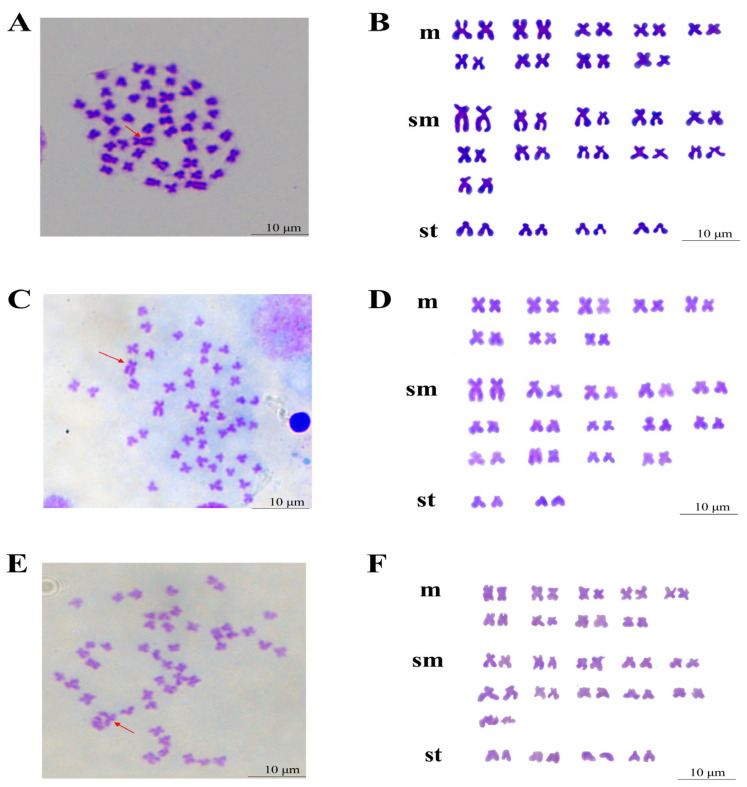
The karyotypes analysis of BG and its parents. (**A**) The chromosomal numbers of BSB. (**B**) The chromosomal karyotype of BSB. (**C**) The chromosomal numbers of GTC. (**D**) The chromosomal karyotype of GTC. (**E**) The chromosomal numbers of BG. (**F**) The chromosomal karyotype of BG. The red arrow indicates the largest submetacentric chromosome. Bar = 10 μm. Note: n = 20 per group.

The chromosome number and karyotype analysis results of BSB, GTC, and BG are shown in Figure 3 and Figure 4.

The results indicated that 85% of BSB cells had 48 chromosomes, following a karyotype formula of 18m + 22sm + 8st (Figure 4A,B). In 82% of GTC cells, there were 48 chromosomes in the division phase, with a karyotype formula of 16m + 28sm + 4st (Figure 4C,D). In 94% of BG cells, there were 48 chromosomes in the division phase, with a karyotype formula of 18m + 22sm + 8st (Figure 4E,F). The chromosome number and karyotype of BG were identical to those of BSB.

### 3.5. Molecular Organization of 5S rDNA and Sox Genes

**Figure 5:**
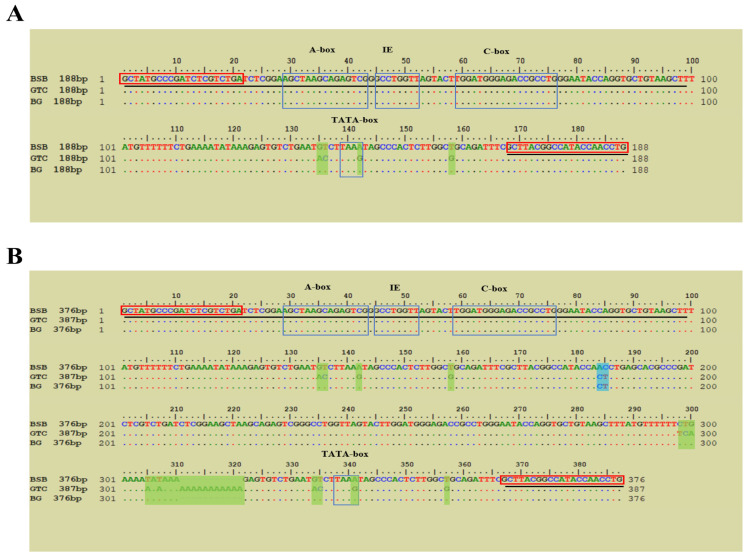
The nucleotide sequence alignment of 5S rDNA fragments in BSB, GTC, and BG. (**A**) Nucleotide sequence alignment of 5S rDNA fragments in BSB (188 bp), GTC (188 bp), and BG (188 bp). (**B**) Nucleotide sequence alignment of 5S rDNA fragments in BSB (376 bp), GTC (387 bp), and BG (376 bp). 5S rRNA is underlined. The blue boxes indicate the regulatory sequences, A-box, intermediate elements, C-box, and TATA-box. The red boxes indicate the PCR primers. The green boxes indicate species-specific regions for BSB and BG. The blue boxes indicate species-specific regions for GTC and BG. Note: n = 20 per group.

Agarose gel electrophoresis revealed that all three fish species exhibited two distinct bands for 5S rDNA (Figure S2), with BG and BSB displaying bands of identical sizes. Sequence analysis showed that the two bands of BSB were 188 bp and 376 bp, the two bands of GTC were 188 bp and 387 bp, and the two bands of BG hybrid fish were 188 bp and 376 bp (Figure 5).

In this study, each 5S rDNA molecule consists of two coding regions (5′-99 bp and 3′-21 bp) separated by an NTS sequence. The NTS sequence of the 188 bp 5S rDNA fragment in BG and BSB was identical, while it differed from that of GTC at positions 135, 136, 143, and 153 (Figure 5A). For the 376 bp 5S rDNA fragment, the NTS sequences in BG and BSB differed at positions 184 and 185, but these positions were identical to those in GTC. However, the 376 bp 5S rDNA NTS sequence in BG and GTC showed differences at multiple positions (Figure 5B).

**Figure 6:**
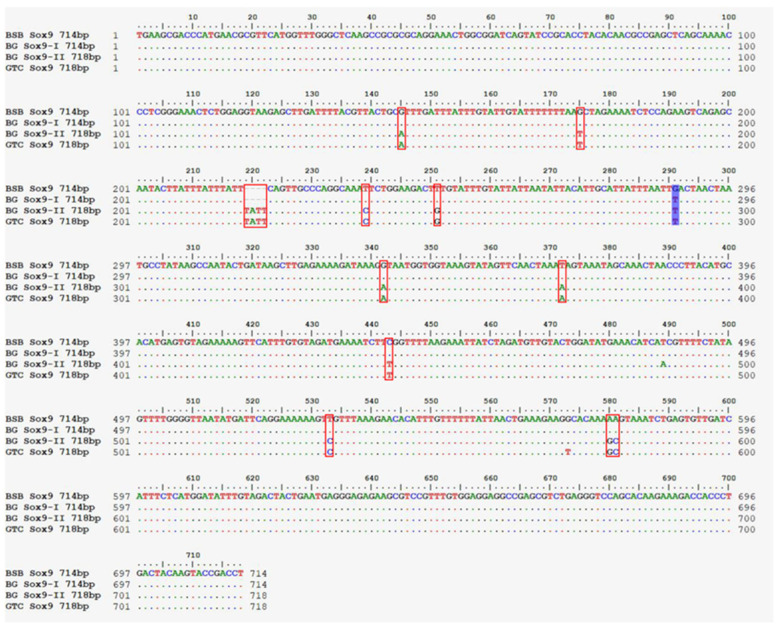
The nucleotide sequence alignment of *Sox9* gene fragments in BSB, GTC, and BG. Nucleotide sequence alignment of *Sox9* gene fragments in BSB (714 bp), GTC (718 bp), BG-I (714 bp), and BG-II (718 bp). The dark purple shadow boxes indicate the bases that are identical in BSB, GTC, and BG. The red boxes indicate the bases with identical sequences in BG and GTC. Note: n = 20 per group.

The gel electrophoresis patterns of amplification products used are shown in Figure S3. BSB, GTC, and the hybrid offspring BG all exhibited two fragments. Detailed results of the sequence analysis, including fragment lengths and gene composition, were provided in the Supplementary Explanation S3. Comparison of the *Sox9* gene sequences from BSB, GTC, BG-I (714 bp), and BG-II (718 bp) (Figure 6) revealed that the 714 bp fragment from BG-I was almost identical to that of BSB. In contrast, the 718 bp fragment from BG-II was nearly identical to that of GTC. Furthermore, both the 714 bp fragment from BG-I and the 718 bp fragment from BG-II exhibited a “T” base at position 291, which was identical to that of GTC.

### 3.6. Mitochondrial Genetic Analysis

**Figure 7:**
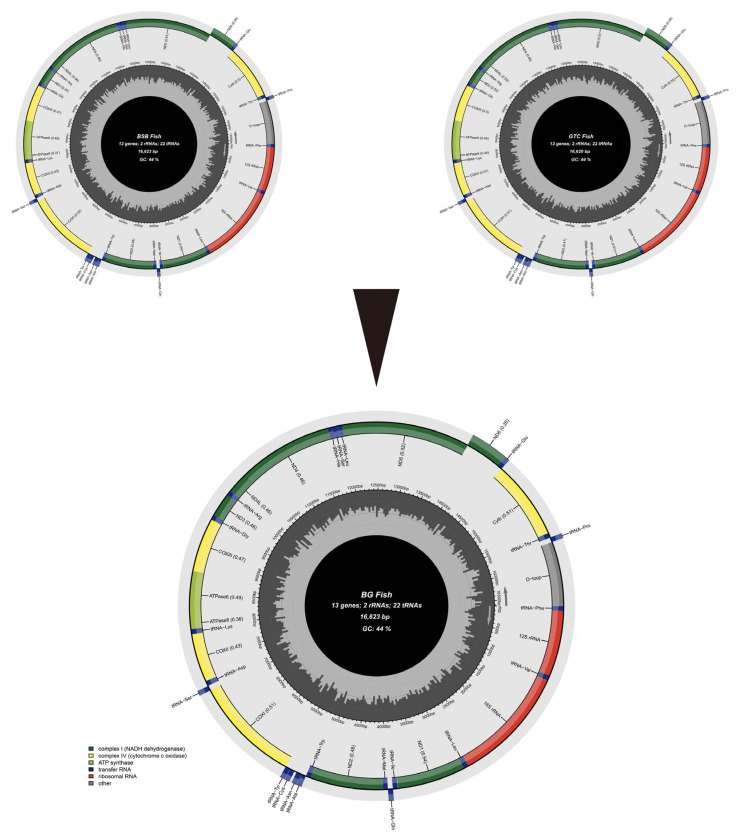
The complete mitochondrial sequences of BSB, GTC, and BG. The gray arrows in the figure indicate the transcriptional orientation of each gene, with those on the heavy strand (inner circle) transcribed clockwise and those on the light strand (outer circle) transcribed counterclockwise.

The results of the assembly and annotation revealed that the complete mitochondrial genome sequences were 16,623 bp for BSB, 16,620 bp for GTC, and 16,623 bp for BG (Figure 7).

Each mitochondrial genome contained 13 protein-coding genes, 22 tRNA genes, and 2 rRNA genes. Specifically, the gene arrangement began with *tRNA-Phe*, followed sequentially by *12S rRNA*, *tRNA-Val*, *16S rRNA*, *ND1*, and extended through to *Cyt b* and *tRNA-Thr*. The sequence alignment results showed that the mitochondrial genome of BG was identical in length to that of BSB, with a homology of 99.69%. The mitochondrial genome of BG differed in length from that of GTC, with a homology of 93.95%. Tables S4 and S5 summarize the specific structural characteristics of the mtDNA in GTC and BG. The length of the *COX1* gene was 1551 bp in BSB, GTC, and BG (Figure S4). The length of the control region (D-loop) for both BG and BSB was 937 bp, while the D-loop of GTC was 936 bp (Figure S5). The analysis of the mitochondrial *COX1* gene and D-loop region revealed that BG’s sequences were identical to those of BSB, with 100% similarity. In contrast, there were significant nucleotide differences in the *COX1* gene and D-loop region sequences between BG and GTC, with similarities of 93.94% and 95.09%, respectively. The base counts and respective base proportions of GTC and BG mtDNA are presented in Table S6.

### 3.7. Growth Performance

**Figure 8:**
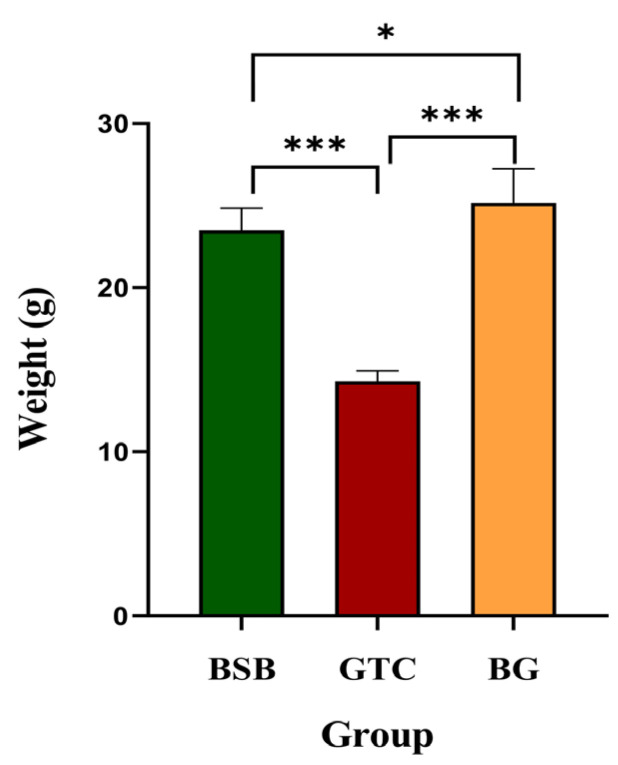
The differences in body weight among 6-month-old BSB, GTC, and BG. “*” indicates a significant difference, * *p* < 0.05, *** *p* < 0.001. Note: n = 20 per group.

The survival rates over the 180-day fattening period are presented in Table S7. Figure 8 illustrates the differences in body weight among 6-month-old BSB, GTC, and BG. The average body weight of the BG hybrid fish reached 25.82 ± 1.50 g, which was significantly greater than that of BSB (22.83 ± 0.28 g, *p* < 0.05). Compared with GTC (14.30 ± 0.62 g, *p* < 0.001), this difference in body weight was even more pronounced, with BG demonstrating an approximately 1.81-fold increase in average body weight relative to GTC. Further heterosis analysis revealed that BG exhibited MPH of 39.19%. Notably, the SPH reached 13.10%.

### 3.8. Fertility Analysis

**Figure 9:**
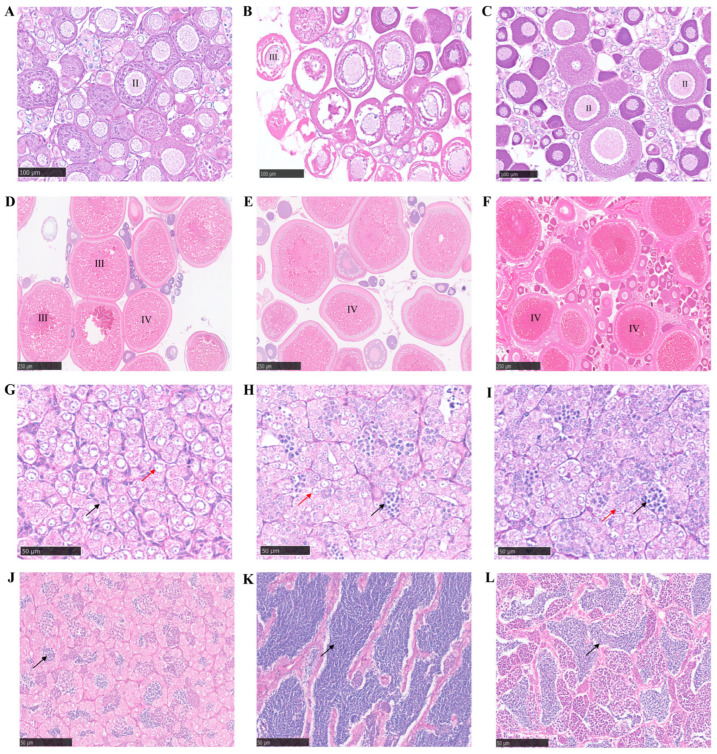
The gonadal microstructure of 6- and 15-month-old BSB, GTC, and BG. (**A**) Ovary of a 6-month-old BSB female with oocytes at stage II. (**B**) Ovary of a 6-month-old GTC female with oocytes at stage III. (**C**) Well-developed ovary of a 6-month-old BG female with oocytes at stage II. (**D**) Ovary of a 15-month-old BSB female with oocytes at stages III and IV. (**E**) Ovary of a 15-month-old GTC female predominantly with oocytes at stage IV. (**F**) Ovary of a 15-month-old BG female with abundant oocytes at stage IV. (**G**) Testis of a 6-month-old BSB male with few spermatids. (**H**) Testis of a 6-month-old GTC male with numerous spermatids. (**I**) Testis of a 6-month-old BG male with abundant spermatids. (**J**) Testis of a 15-month-old BSB male with a few mature spermatozoa. (**K**) Seminiferous tubules of a 15-month-old GTC male testis filled with mature spermatozoa. (**L**) Seminiferous tubules of a 15-month-old BG male testis with abundant mature spermatozoa. Red arrows: spermatogonia; black arrows: spermatids. Note: n = 20 per group.

The gonadal development of BSB, GTC, and BG at 6 and 15 months of age are shown in Figure 9. At 6 months, the ovaries of BSB remained at Stage II, while the testis contained sparse spermatids (Figure 9A,G). In contrast, GTC exhibited higher synchronicity in ovarian development, with ovaries at Stage III, and the testis showed abundant clusters of spermatids (Figure 9B,H). The ovarian development of BG remained at Stage II, and spermatogonia and spermatids were observed within the seminiferous tubules of the testis (Figure 9C,I). By 15 months, BSB ovaries were dominated by Stage III and IV oocytes, with the testis containing some mature spermatozoa (Figure 9D,J). In GTC, the ovaries were predominantly composed of Stage IV oocytes, and the seminiferous tubules of the testis were densely packed with mature spermatozoa (Figure 9E,K). Meanwhile, the ovaries of BG were also filled with Stage IV oocytes, males were capable of releasing milky semen, and the seminiferous tubules contained tightly arranged mature spermatozoa (Figure 9F,L).

### 3.9. Preliminary Analysis of Nutritional Components in the Muscle of BG and Its Parents

The crude protein and fat contents of muscle in BSB, GTC, and BG are shown in Table 4.

The crude protein content of BG was slightly higher than that in BSB and GTC (both *p* > 0.05). Effect size analysis (Hedges’ g) showed that the comparative effect values were 0.369 for BSB versus BG and 0.246 for GTC versus BG, both indicating a small effect size. The findings demonstrated that there was a consistent slight increasing trend in crude protein content in BG compared to its parents. On the contrary, its crude fat content was significantly lower than that of BSB (*p* < 0.01) and GTC (*p* < 0.05).

The amino acid contents of muscle in BSB, GTC, and BG are shown in Table 5. Seventeen hydrolyzed amino acids, including seven essential amino acids and ten non-essential amino acids, were detected in both BG and its parents. Compared with BSB, BG exhibited higher content in all amino acids except for tyrosine (Tyr), while its overall amino acid profile was generally similar to that of GTC. Notably, the total umami amino acid content in BG muscle was higher than that in BSB and slightly exceeded that in GTC. Moreover, the overall amino acid content in BG surpassed that of both parents. The ratio of essential amino acids to total amino acids in the muscles of BG, BSB, and GTC consistently fell within a narrow range of 40~42%, with minimal variation. The fatty acid contents of the muscle in BSB, GTC, and BG are shown in Table S8. In the muscle tissues of BG and its parents, 14 fatty acids were detected, including 3 saturated fatty acids and 11 unsaturated fatty acids. Compared to the BSB, BG showed lower levels in all individual fatty acids except for eicosapentaenoic acid (C20:5n3, EPA) and docosahexaenoic acid (C22:6n3, DHA). Both the monounsaturated fatty acid and the total fatty acid contents in BG were significantly lower than those in BSB (*p* < 0.01). Relative to the GTC, BG exhibited higher levels only in eicosadienoic acid (C20:2, EDA) and DHA, while the remaining fatty acid components were present at lower concentrations. Notably, the total polyunsaturated fatty acid content in BG was higher than that in GTC.

The amino acid content (mg/g N), AAS, CS, and EAAI of BSB, GTC, and BG are shown in Table S9. The essential amino acid (EAA) content of BG muscle was 2253.72 mg/g N, which was comparable to the FAO/WHO standard of 2190 mg/g N but significantly lower than the whole egg protein standard of 2959 mg/g N. In contrast, the lysine (Lys) content in BG muscle exceeded both reference values. With the exception of methionine + cysteine (Met + Cys), the AASs of other essential amino acids in BG muscle were all above 0.80, and the CSs were all above 0.60. Based on the AASs and CSs, the first and second limiting amino acids in BG muscle were Met + Cys and valine (Val), respectively. Moreover, the essential amino acid index (EAAI) of BG muscle was higher than that of BSB.

## 4. Discussion

In recent years, the degradation of germplasm and the increasing scarcity of elite germplasm in *M. amblycephala* have impeded the development of its large-scale aquaculture. Although migration-based restoration serves as a primary strategy for conserving wild populations and biodiversity, controlled hybridization has been widely applied in aquaculture as a distinct approach aimed at enhancing production-oriented traits [28]. The distant hybridization of fish is an essential technique in fish genetics and breeding. By integrating the advantages of parents, the traits of hybrid offspring can be improved, and offspring with advantages in growth, stress resistance, and quality can be produced [29]. Distant hybridization has facilitated the establishment of novel fish lineages that exhibit genetic variation, fertility, and enhanced characteristics. This approach has also had a profound impact on the advancement of fish breeding technologies and the growth of the aquaculture industry [30].

At present, a relatively large number of distant hybridizations are associated with *M. amblycephala*, which can be fertilized and produce viable offspring [31]. Diploid and triploid progeny have been successfully obtained from distant hybridization between *M. amblycephala* (♀) and *X*. *davidi* (♂). The hybrid offspring had the advantages of a high fertilization and hatching rate, good shape, tender meat, strong resistance, and so on [32]. The researchers successfully obtained triploid offspring by crossing *M. amblycephala* (♀) × *C. alburnus* (♂). The triploid gonads were underdeveloped or showed signs of degeneration and more energy was used for growth [33]. In this study, a novel intergeneric hybrid fish (BG) was successfully generated through distant hybridization between BSB and GTC, exhibiting favorable phenotypic traits. The ploidy and chromosome analyses confirmed that BG was a diploid hybrid. The hybrid demonstrated remarkable developmental advantages, showing a shorter embryonic developmental period than BSB under water temperatures of 25~27 °C. This advantage, along with markedly improved fertilization and hatching rates, indicating that distant hybridization effectively enhanced the reproductive performance of the offspring. Morphometric analysis further revealed that most growth traits of BG were intermediate between the two parental species, displaying typical heterosis. A pivotal advance of this study over previous distant hybridization in bream is the development of BG, a stable diploid hybrid. This hybrid exhibits typical heterosis in growth and morphology, coupled with a significantly accelerated embryonic developmental rate. This advantage suggests the potential for a shorter growth cycle at the seedling stage. Collectively, these attributes offer irreplaceable core value for this new germplasm resource.

In this study, the growth performance of BG hybrid fish was compared with that of its parents (Figure 8). The experimental results demonstrated that, after a 180-day culture period, the average body weight of the BG exceeded that of both parents, reaching 1.81-fold that of its male parent, GTC. The improved growth performance of BG holds significant implications for enhancing yield and economic efficiency in aquaculture. Despite the overall low survival rates observed across all groups, which may be attributed to prolonged tank environmental stress, the growth performance data remain valid and informative. A limitation of this study is that the growth trials were conducted in one facility under controlled conditions; thus, environmental effects could not be fully evaluated. However, to comprehensively evaluate its growth performance and application value, further long-term systematic breeding experiments and detailed economic benefit analyses are required.

As a practical genomic DNA marker, 5S rDNA exhibits species-specific characteristics and has commonly been used to understand variation, evolution, and genetic relationships among species [22,34]. In this study, purification and sequencing analysis revealed that the band sizes in BG were consistent with those of the maternal parent BSB (class I: 188 bp, class II: 376 bp). Sequence alignment showed that the 188 bp 5S rDNA sequence in BG was identical to that of BSB (Figure 5A). However, the 376 bp 5S rDNA sequence had two nucleotide differences in the NTS region compared to BSB, but these sites were identical to those in the paternal parent GTC (Figure 5B). The presence of both parental alleles at distinct, unlinked loci in BG serves as supporting evidence that it originated from hybridization between BSB and GTC.

The *Sox* genes family plays a critical role in various biological processes, including sex determination, organogenesis, neural development, cell fate specification, and embryonic patterning. *Sox* transcription factors are central to the development of multiple organs, particularly the gonads, heart, and pancreas [35,36]. Notably, the *Sox9* gene has been shown to have a direct role in sex determination in vertebrates, making it a key focus in the study of sex differentiation mechanisms [37]. In this study, the *Sox9*-714 bp and *Sox9*-718 bp fragment analysis revealed that these two fragments showed high homology to the *Sox9*-714 bp from BSB and the *Sox9*-718 bp from GTC, respectively, indicating that BG inherited genetic material from both parents (Figure 6). In addition, both fragments contained a “T” base at position 291, identical to that of GTC. These results suggest that BG may have inherited more genetic information related to gonadal development from the paternal parent GTC.

In early research, mtDNA was primarily used as a molecular marker, particularly specific sequences or genes of mtDNA. For instance, the mtDNA D-loop region was used to analyze the evolution of different populations within the same species. Meanwhile, genes such as *Cytb*, *COX1*, 16S, and 12S rRNA served as vital biological markers for species identification [38,39]. Researchers have assessed phylogenetic relationships and maternal origins of different fish populations by analyzing the mtDNA D-loop region and *COX1* gene [40]. In this study, the full-length mitochondrial gene of BG was identical to that of BSB, with a homology of 99.69%. Annotation of mitochondrial DNA genes revealed that the mitochondrial structure of BG was also identical to that of BSB. Comparative analysis of the mitochondrial *COX1* gene and D-loop region sequences showed that BG and BSB had identical sequences for both markers. This further confirmed the maternal inheritance characteristic of BG mitochondria.

In the distant hybridization of fish, the large number of fish species and their relatively low degree of genetic differentiation are common. These factors significantly increase the possibility of forming fertile strains through distant hybridization [17]. Fertile hybrids produced by BSB and TC in our laboratory enabled the successful establishment of hybrid lines BTF_1_~BTF_6_ and TBF_1_~TBF_3_ [14,41,42]. However, in most cases, the F_1_ generation produced by the distant hybridization of fish is less fertile or sterile. In the diploid hybrid F1 from *M. amblycephala* (♀) × *X. davidi* (♂), the male individuals were found to have poorly developed testes. In contrast, the female individuals lacked any apparent ovarian structure. Gonadal tissue slices showed that some spermatozoa in the testes had developed into sperm [32]. During the breeding season, males can extrude a small amount of watery semen, which can be used to produce offspring, but females do not produce normally developed eggs. In this study, histological analysis of gonadal tissues results demonstrated that spermatogenesis proceeds more rapidly in BG than in BSB. This finding suggested that BG may offer advantages for improving germplasm utilization efficiency and shortening the breeding cycle. Notably, the fertility of BG hybrids likely stems from chromosomal compatibility and ploidy stability between the parent species, which facilitated normal meiotic progression and viable gamete formation. The hybrid genome maintains a stable diploid, allowing for proper chromosome pairing and segregation during gametogenesis. In addition, during the breeding season of 2024, two-year-old BG individuals could produce white sperm or mature eggs and provide a large number of offspring through self-crossing and back-crossing. Thereby, they overcame the transgenerational bottleneck of sterility that is typically observed in triploid hybrids.

The nutritional value of fish was primarily determined by the protein and fat content in their muscle. Previous studies have demonstrated that hybridization can produce progeny with a superior muscle nutritional composition compared to that of their parental species. For instance, two improved hybrid breams, derived from a hybrid lineage of *M. amblycephala* (♀) × *C. alburnus* (♂), exhibited outstanding muscle nutritional characteristics [14]. Essential amino acids (EAA) are critical indicators of the nutritional value of proteins in food, as they must be obtained from external dietary sources [43]. In this study, the comparative analysis revealed that the total EAA content was higher in BG than in BSB. Moreover, the composition and concentration of umami amino acids are key determinants of fish flavor profile, among which glutamic acid (Glu) is regarded as the predominant contributor [44]. The total umami amino acid content in BG muscle was determined to be 5.45%, surpassing levels in BSB and GTC. Notably, the Glu content was significantly higher than in the parents. These findings suggest that BG may exhibit superior flavor attributes. Further evaluation showed that the ∑EAA/∑TAA ratio in BG muscle exceeded the FAO/WHO reference pattern for high-quality protein, thereby classifying it as a high-quality dietary protein source. Assessment based on the AAS, CS, and EAAI [45] identified Met + Cys and Val as the first and second limiting amino acids in BG, respectively. The EAAI results further indicate that BG possesses a higher amino acid nutritional value compared to BSB, implying the potential for improved muscle quality.

The nutritional value of lipids is primarily determined by the types and amounts of fatty acids they contain, particularly the levels of unsaturated fatty acids [46]. In this study, according to Table S8, the saturated fatty acid (SFA) content in BG muscle was lower than that of its parents. In contrast, the unsaturated fatty acid (UFA) content was significantly lower than that of BSB (*p* < 0.01), but comparable to GTC. Regarding nutritionally important polyunsaturated fatty acids (PUFA), the content in BG was 0.1346%, which was higher than that in GTC. Notably, the levels of long-chain n-3 polyunsaturated fatty acids (n-3 PUFA) in BG muscle, including eicosapentaenoic acid (EPA, C20:5n3) and docosahexaenoic acid (DHA, C22:6n3), were higher than those in its parents. These two n-3 PUFAs are widely recognized for their significant physiological functions in preventing atherosclerosis, thrombosis, and hypertension [47,48]. Therefore, we speculate that BG may contain higher levels of polyunsaturated fatty acids beneficial to human health. The differences in fatty acid content between BG and its parents might reflect distinct fatty acid metabolic pathways, likely related to the interactions and genetic variations of heterozygous genes in BSB and GTC [27].

## 5. Conclusions

In this study, we successfully generated a novel, fertile diploid hybrid fish (BG) via intergeneric hybridization between female BSB and male GTC. A genetic analysis based on 5S rDNA and mtDNA sequences indicated that BG inherited a predominantly maternal (BSB) genomic profile, while also incorporating specific paternal (GTC) DNA fragments. Furthermore, the detection of paternal sequences, including partial 5S rDNA class II and the *Sox9*-718 bp fragment, provided direct evidence of the complex interactions between these heterologous genomes. The histological examination confirmed that BG possessed bisexual fertility, enabling the production of viable offspring through both self-crossing and back-crossing at two years of age. Furthermore, the nutritional analysis demonstrated that BG muscle has enhanced protein quality and a favorable lipid composition compared to its parents. With its stable inheritance, reproductive capacity, and superior nutritional attributes, BG represents a valuable novel germplasm resource, indicating potential value for selective breeding programs. In the upcoming research, further multi-generation studies are needed to assess the genetic stability and production potential of this hybrid.

## Figures and Tables

**Table 1:** Comparison of the measurable morphological traits of blunt snout bream (BSB), green tip culter (GTC), and BG.

Fish Type	WL/BL	BL/BH	BL/HL	HL/HH	BH/HH	CPL/CPH
BSB	1.24 ± 0.01	2.39 ± 0.02 ^##^	4.42 ± 0.14 ^#^	1.27 ± 0.04 ^#^	2.35 ± 0.11 ^###^	1.25 ± 0.01
GTC	1.24 ± 0.03	4.13 ± 0.17 **	3.74 ± 0.20 *	1.53 ± 0.08 *	1.38 ± 0.07 ***	1.82 ± 0.25
BG	1.22 ± 0.01	2.98 ± 0.03 ***^,##^	4.24 ± 0.16 ^#^	1.34 ± 0.10	1.91 ± 0.13 *^,##^	1.43 ± 0.05 *

“*” indicates a significant difference from BSB, * *p* < 0.05, ** *p* < 0.01, *** *p* < 0.001; “^#^” indicates a significant difference from GTC, ^#^ *p* < 0.05, ^##^ *p* < 0.01, ^###^ *p* < 0.001. The following table is the same. Note: Data are presented as mean ± standard deviation (SD) from each group (n = 20).

**Table 2:** Comparison of the countable morphological traits of BSB, GTC, and BG.

Fish Type	Number of lateral Scales	Number of Upper lateral Scales	Number of Lower Lateral Scales	Number of Dorsal Fins ^a^	Number of Abdominal Fins	Number of Anal Fins ^a^
BSB	50~57	12	8~9	III + 7~8	8~9	III + 25~27
GTC	62~70	12~13	7~8	III + 8	9	III + 22~27
BG	54~61	12~13	8~9	III + 8	9~10	III + 25~27

^a^: “III” represents hard fin rays and Arabic numerals represent soft fin rays. Note: Data are presented as the observed range from each group (n = 20).

**Table 3:** The average DNA content of BSB, GTC, and BG.

Fish Type	Average DNA Content	Ratio	
		Observed	Expected
BSB	69.48		
GTC	69.12		
BG	68.12	BG/BSB = 0.98 BG/GTC = 0.99	1

**Table 4:** The crude protein and crude fat content of muscle in BSB, GTC, and BG.

Fish Type	Crude Protein (g/100 g)	Crude Fat (g/100 g)
BSB	17.83 ± 0.35	1.27 ± 0.06
GTC	17.93 ± 0.35	1.10 ± 0.10
BG	18.13 ± 0.85	0.87 ± 0.06 **^,#^

“*” indicates a significant difference from BSB, ** *p* < 0.01; “^#^” indicates a significant difference from GTC, ^#^ *p* < 0.05. Note: Data are presented as the means ± SD, n = 9 replicates per group.

**Table 5:** Amino acids contents of BSB, GTC, and BG.

Amino Acid Types	BSB	GTC	BG
Asp **^b^** (g/100 g)	1.47 ± 0.06	1.54 ± 0.11	1.54 ± 0.17
Thr **^a^** (g/100 g)	0.66 ± 0.02	0.67 ± 0.03	0.68 ± 0.06
Ser (g/100 g)	0.54 ± 0.03	0.57 ± 0.05	0.57 ± 0.07
Glu **^b^** (g/100 g)	2.04 ± 0.11	2.12 ± 0.18	2.15 ± 0.28
Gly **^b^** (g/100 g)	0.79 ± 0.03	0.79 ± 0.02	0.80 ± 0.12
Ala **^b^** (g/100 g)	0.94 ± 0.02	0.98 ± 0.04	0.96 ± 0.09
Cys (g/100 g)	0.11 ± 0.02	0.11 ± 0.00	0.11 ± 0.01
Val **^a^** (g/100 g)	0.75 ± 0.02	0.77 ± 0.03	0.77 ± 0.06
Met **^a^** (g/100 g)	0.31 ± 0.02	0.33 ± 0.04	0.33 ± 0.07
Ile **^a^** (g/100 g)	0.67 ± 0.03	0.69 ± 0.03	0.68 ± 0.06
Leu **^a^** (g/100 g)	1.25 ± 0.02	1.27 ± 0.04	1.27 ± 0.10
Tyr (g/100 g)	0.50 ± 0.03	0.50 ± 0.02	0.49 ± 0.03
Phe **^a^** (g/100 g)	0.66 ± 0.02	0.68 ± 0.05	0.67 ± 0.08
Lys **^a^** (g/100 g)	1.51 ± 0.03	1.54 ± 0.07	1.53 ± 0.14
His (g/100 g)	0.35 ± 0.00	0.38 ± 0.01	0.41 ± 0.04
Arg (g/100 g)	0.90 ± 0.02	0.91 ± 0.02	0.91 ± 0.08
Pro (g/100 g)	0.47 ± 0.02	0.47 ± 0.01	0.48 ± 0.05
∑DAA	5.25 ± 0.20	5.43 ± 0.33	5.45 ± 0.64
∑EAA	5.81 ± 0.05	5.96 ± 0.26	5.94 ± 0.55
∑TAA	13.93 ± 0.21	14.33 ± 0.67	14.37 ± 1.46
∑DAA/∑TAA	0.38 ± 0.01	0.38 ± 0.01	0.38 ± 0.01
∑EAA/∑TAA	0.42 ± 0.01	0.41 ± 0.01	0.41 ± 0.01

“^a^” indicated essential amino acids; “^b^” indicated umami amino acids. DAA: umami amino acids; EAA: essential amino acids; TAA: total amino acids. Note: Data are presented as the means ± SD, n = 9 replicates per group.

## Data Availability

Data will be made available on request.

## Supplementary Materials

The following supporting information can be downloaded at: https://www.mdpi.com/article/10.3390/ani15223302/s1, Figure S1: The embryonic development of BG; Figure S2: The PCR amplification map of 5S rDNA among BSB, GTC, and BG; Figure S3: The gel electrophoresis profiles of BSB, GTC, and BG Sox genes amplification; Figure S4: The sequence of the cytochrome c oxidase subunit I (COX1) gene in BSB, GTC, and BG; Figure S5: The sequence of the control region (D-loop) in BSB, GTC, and BG. Table S1: The mitochondrial full-length amplification primer sequence of GTC and BG; Table S2: The length of time after fertilization at different developmental stages of BG; Table S3: The fertilization rates, hatching rates, and Hatching period of BSB, GTC and BG hybrids; Table S4: The specific structural features of the mitochondrial DNA of GTC; Table S5: The specific structural features of the mitochondrial DNA of BG; Table S6: The proportions and counts of bases in GTC and BG mitochondrial DNA; Table S7: The survival rates throughout the 180-day fattening period; Table S8: Fatty acid content of BSB, GTC, and BG; Table S9: Amino acid content (mg/g N), AAS, CS and EAAI of BSB, GTC, and BG. Supplementary Explanation S1: Supplementary notes for Figure 5; Supplementary Explanation S2: Supplementary notes for Figure 6; Supplementary Explanation S3: Supplementary analysis of the Sox genes; Supplementary Explanation S4: Supplementary of water quality logs and food composition|ratios; Supplementary Explanation S5: Supplementary of the formula for calculating protein nutritional index. Supplementary Explanation S6: Supplementary of detailed information for the two standards: GB 5009.5-2016 and GB 5009.6-2016.
