# Supplementary material for: The Formation of a Novel Intergeneric Hybrid Fish Derived from Megalobrama amblycephala (♀) × Culter dabryi (♂)
Source: Animals (Basel). 2025 Nov 15;15(22):3302. doi: 10.3390/ani15223302 (PMC12649757; doi:10.3390/ani15223302)
Supplement: Supplementary file 1 [file animals-15-03302-s001.zip › animals-3909924-supplementary.pdf]

Figure S1 The embryonic development of BG.

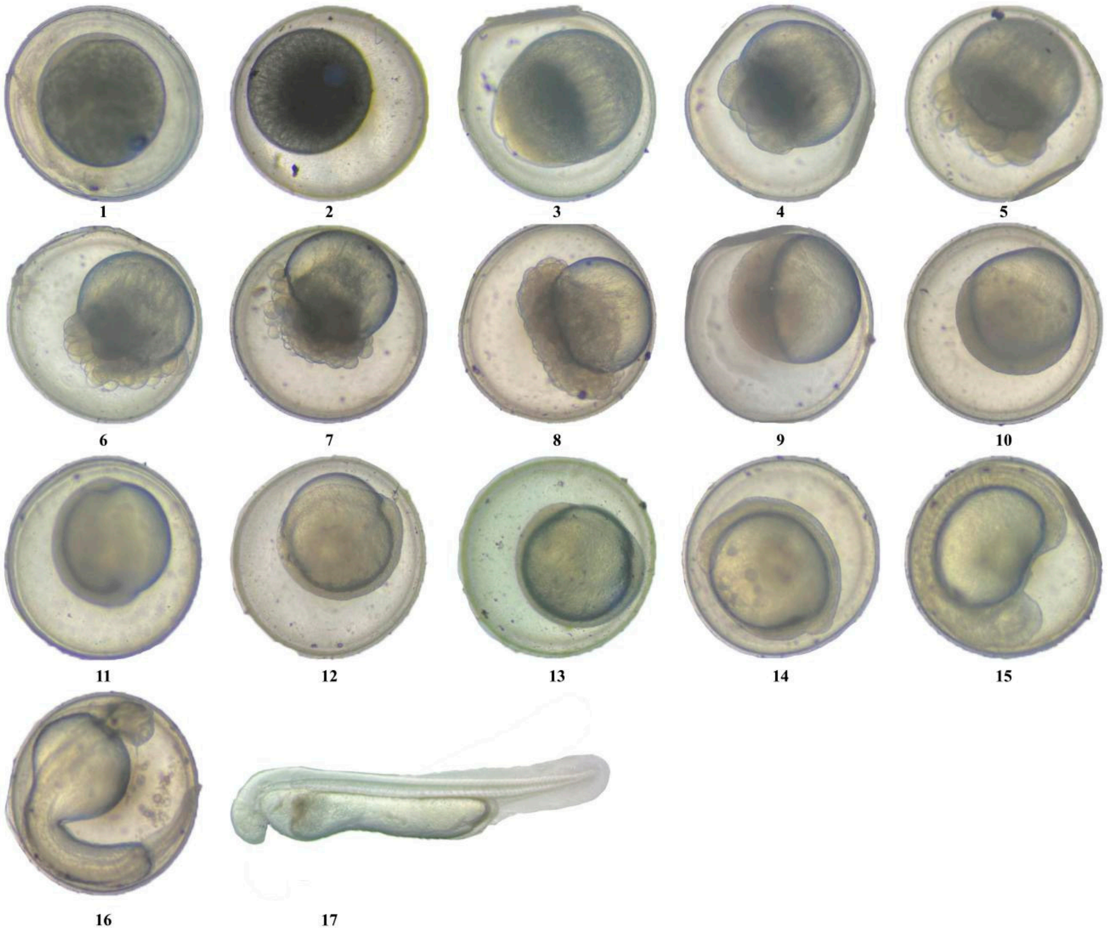

1. Fertilized egg stage; 2. Embryo disk stage; 3. 2-cell stage; 4.4-cell stage; 5. 16-cell stage; 6. 32-cell stage; 7. 128-cell stage; 8. Mulberry embryo stage; 9. Blastocyst stage; 10. Early grastrula stage; 11. Mid-gastrula stage; 12. Late gastrula stage; 13. Neurula stage; 14. Sarcomere appearance stage; 15. Occurrence stage of caudal fin; 16. Muscle pulsation stage; 17. Larval stage.

Figure S2 The PCR amplification map of 5S rDNA among BSB, GTC, and BG.

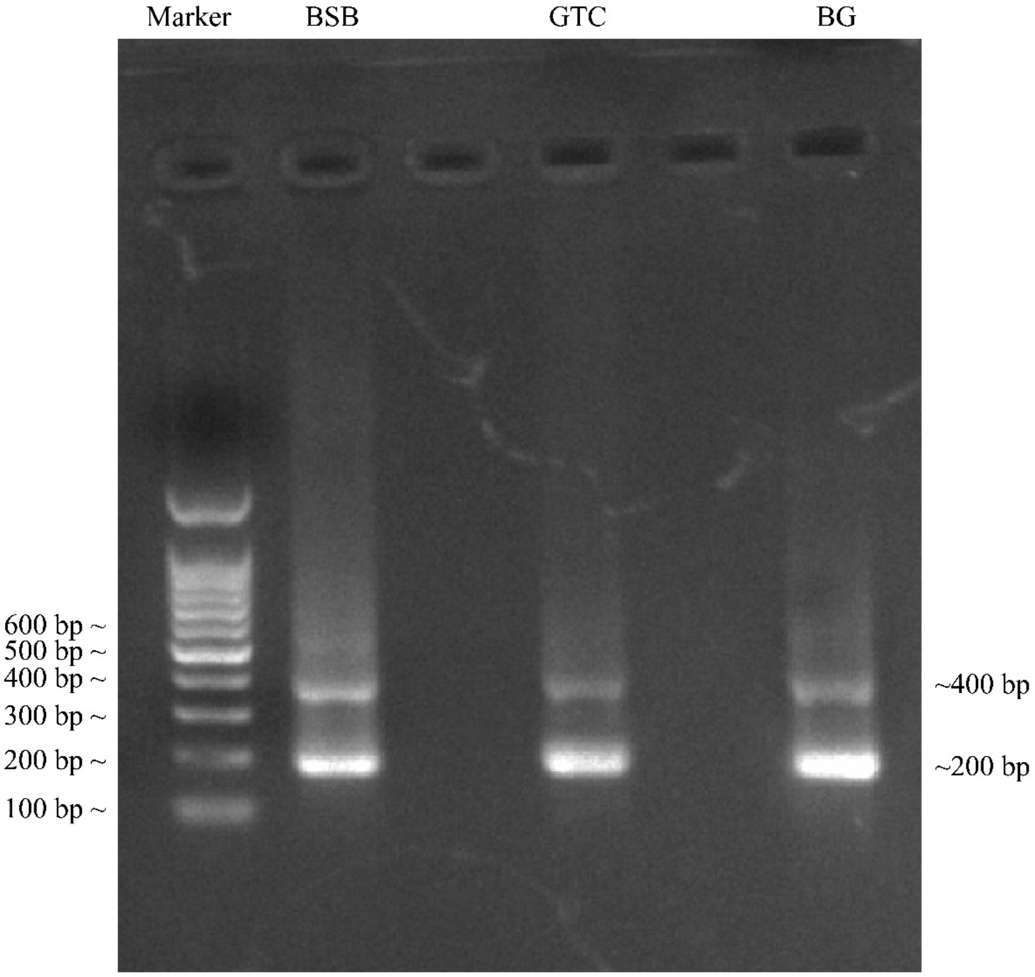

DNA bands of 5S rDNA amplified from BSB, GTC and BG hybrids. Marker: 100 bp DNA ladder; BSB: two DNA bands (~200 and ~ 400 bp); GTC: two DNA bands (~200 and ~ 400 bp); and BG: two DNA bands (~200 and ~ 400 bp). Note: n = 20 per group.

Figure S3 The gel electrophoresis profiles of BSB, GTC, and BG *Sox* genes amplification.

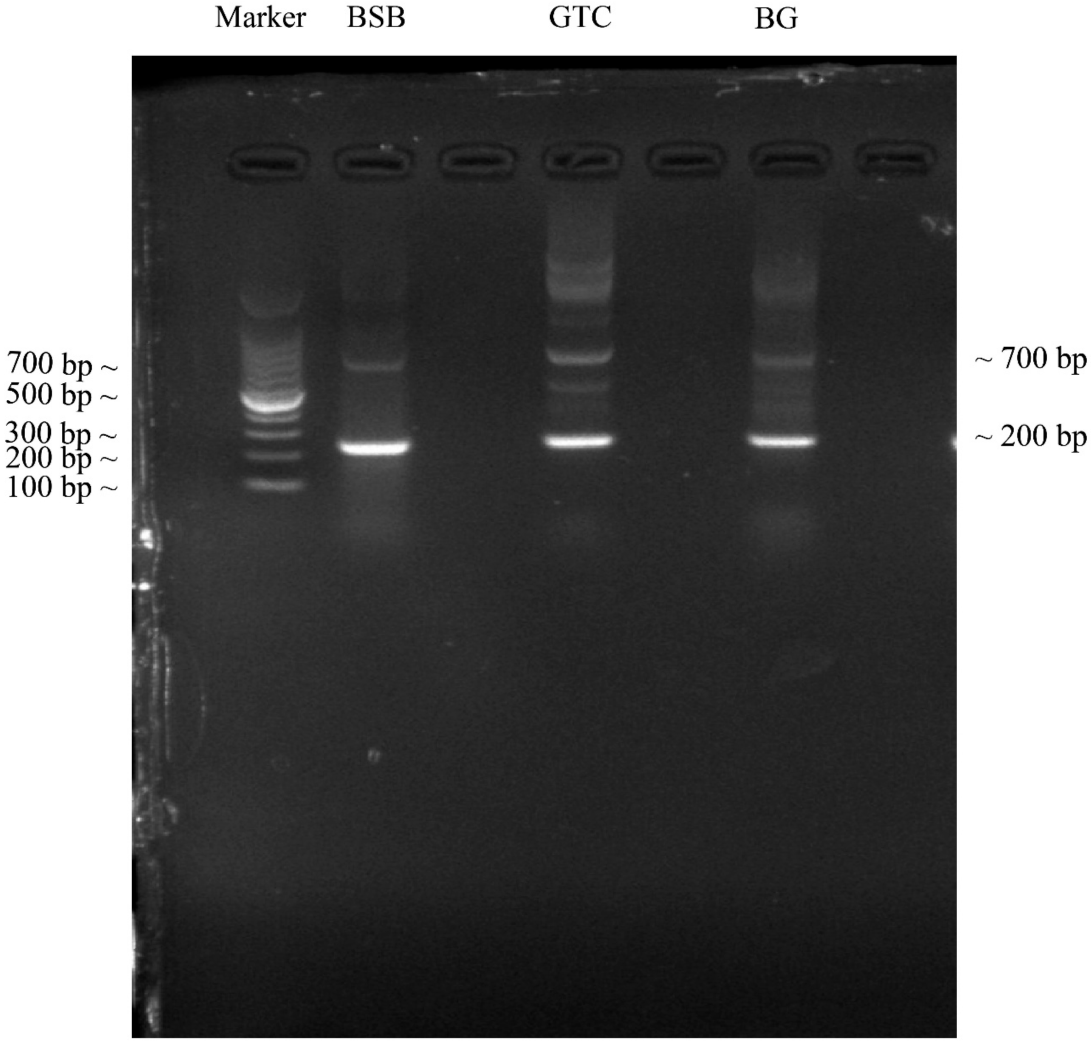

DNA bands of *Sox* genes amplified from BSB, GTC, and BG hybrids. Marker: 100 bp DNA ladder; BSB: two DNA bands (~200 and ~ 700 bp); GTC: two DNA bands (~200 and ~ 700 bp); and BG: two DNA bands (~200 and ~ 700 bp). Note: n = 20 per group.

Figure S4 The sequence of the cytochrome c oxidase subunit I (*COX1*) gene in BSB, GTC, and BG.

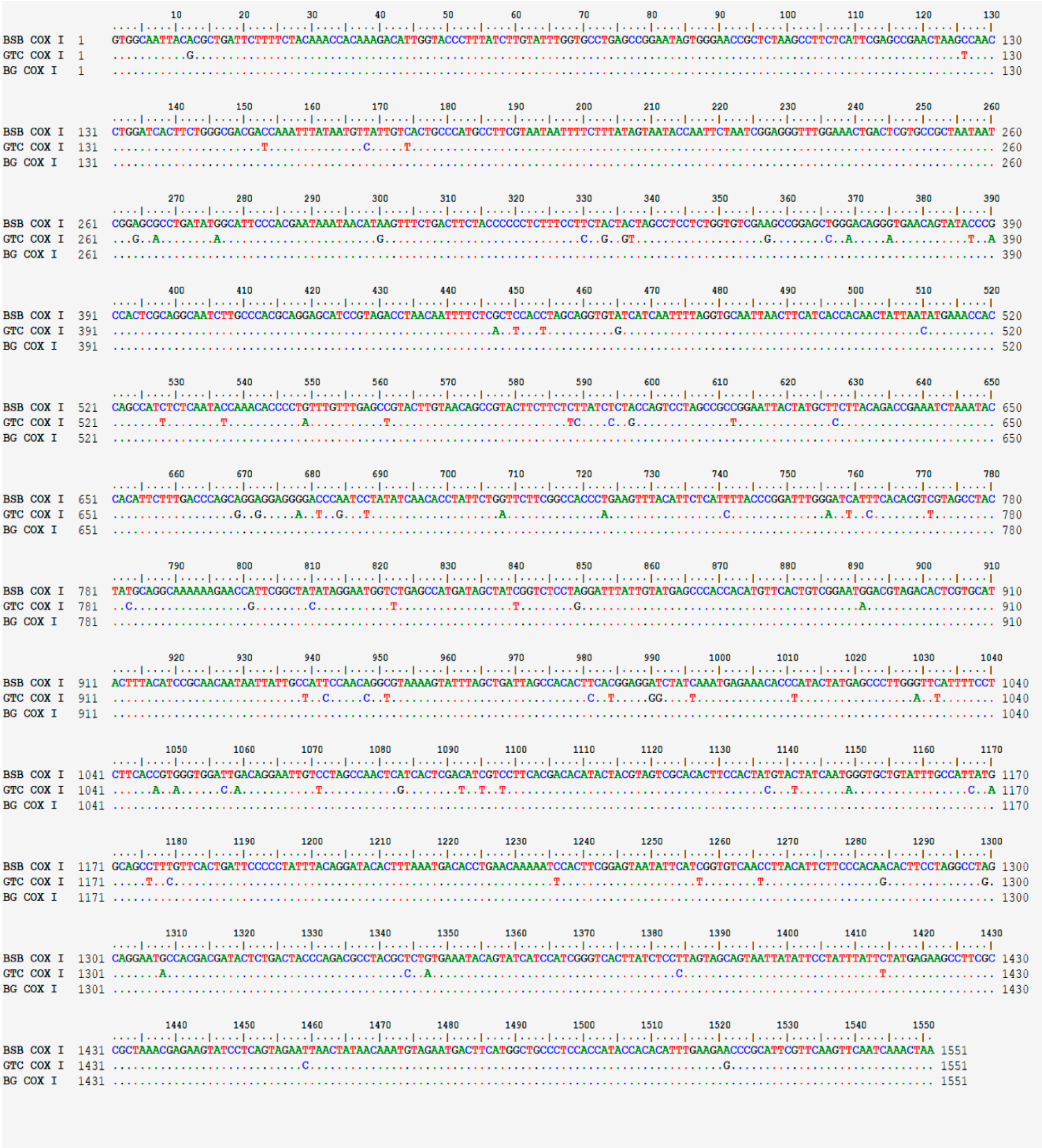

Nucleotide sequence alignment of *COX1* gene fragments in BSB (1551 bp), GTC (1551 bp) and BG (1551 bp).

Figure S5 The sequence of the control region (D-loop) in BSB, GTC, and BG.

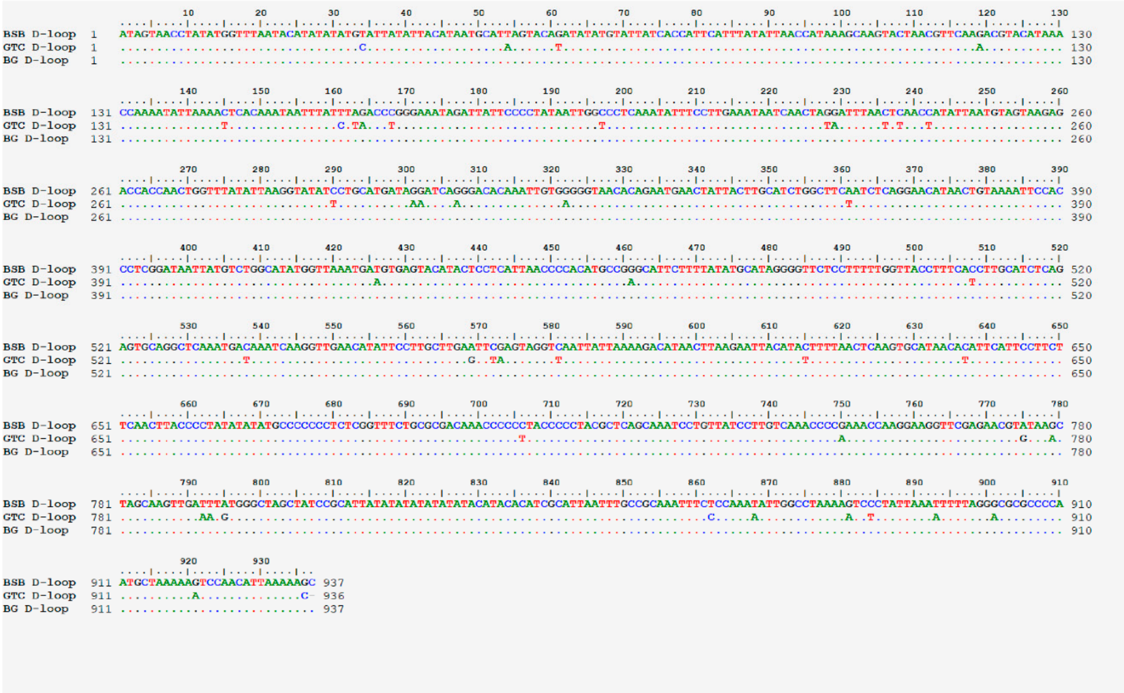

Nucleotide sequence alignment of D-Loop fragments in BSB (937 bp), GTC (936 bp), and BG (937 bp).

Table S1 The mitochondrial full-length amplification primer sequence of GTC and BG.

Primers	Forward primer sequences (5′-3′)	Reverse primer sequences (5′-3′)
MT-1[@]	AACAAAGCCCACCTGACC	CGAGGCTGAACTTCTATCC
MT-2[@]	CTACCGAACCTGGTGATA	AAACTGACCTGGATTGCT
MT-3[@]	GGAGCAATCCAGGTCAGT	ATTGATGGAGGAGGGACT
MT-4[@]	CGAATGCTTAGGGACCAC	AAGGCTCTTGGTCTGATG
MT-5[@]	ATCCTCCCTCCCACTCAC	GTGGTGGGCTCATACAAT
MT-6[@]	TTGCTCTGCCATCCCTTC	GCTGTGGCGATGAGTTGA
MT-7[@]	AGGACATAAATGGGCACT	GAAATGATGTTCGGATGTA
MT-8[@]	GGCTGTCTGTCTTCTTCG	TGAGGCGTTCAGTTTGGT
MT-9[@]	CACCACGCAACATAGAAA	AGGAAGTGGAATGCGAAG
MT-10[@]	TCGCATTCCACTTCCTAC	AGCCTGCACTCTGAGATG
MT-11[*]	GGGTTCTCCTTTTTGGTTAC	TGTTTCTCAGTTTTCGTGG
MT-12[*]	CTACTACGCAGGCAAAAAAG	CATTGATGTCCTATGGCTTT
MT-13[*]	TCACACGATGAGGAAACC	TGACGGCAGTTTTGACAT
MT-14[*]	CAGTTCATCCATTGGTCTTA	TAGTTGCGGTTGGTTTAGTC
MT-15[*]	TAGCATTCCTCCACATCTG	TTAGGTGTTGTATTAGCGG
MT-16[#]	TGCATCTGGCTTCAATCT	ATGGCTGAGCATAGTGGG
MT-17[#]	TTCTTTGACCCAGCAGGAG	GAAGGGATGGCAGAGCAA
MT-18[#]	AACCTATCAGCCGACAAC	AATAGGTGTTCTCGGGTG
MT-19[#]	ACACCCGAGAACACCTAT	GGCTGTGAATAAGGTGGT
MT-20[#]	TCCTACTAATCCGCCTCC	TTCTATGTTGCGTGGTGA

"@" represented the universal primer for both amplifications, "*" represented the specific primer used for GTC amplification, and "#" represented the specific primer used for BG amplification.

Table S2 The length of time after fertilization at different developmental stages of BG.

Developmental stages	Time /(min)	T/ (℃)	Developmental stages	Time /(min)	T/ (℃)
Fertilized egg stage	0	25	Late gastrula stage	6h 23min	26
Embryo disk stage	20min	25	Neurula stage	7h 13min	26
2-cell stage	40min	25	Sarcomere appearance stage	9h 54min	27
4-cell stage	50min	25	Occurrence stage of caudal fin	12h 55min	26
16-cell stage	1h 11min	26	Muscle pulsation stage	14h 56min	26
32-cell stage	1h 22min	26	Larval stage	31h 44min	27
128-cell stage	1h 38min	26			
Mulberry embryo stage	2h 21min	26			
Blastocyst stage	4h 18min	26			
Early grastrula stage	5h 6min	26			
Mid-gastrula stage	5h 52min	26			

Table S3 The fertilization rates, hatching rates, and Hatching period of BSB, GTC and BG hybrids.

Fish Group	Fertilization rate (%)	Hatching rate (%)	Hatching period (h)
BSB (♀) × BSB (♂)	81.67 ± 1.10	$73.83 \pm 0.91^{\#\#}$	33~35
GTC (♀) × GTC (♂)	80.07 ± 0.35	$79.43 \pm 1.10^{**}$	31~34
BSB (♀) × GTC (♂)	$87.80 \pm 1.85^{*\#}$	$80.57 \pm 1.90^{*}$	31~33

"*" indicates a significant difference from BSB, * $p < 0.05$, ** $p < 0.01$; "#" indicates a significant difference from GTC, # $p < 0.05$, ## $p < 0.01$. Data are presented as mean ± standard deviation (SD) from each group (n = 10 replicates per group).

Table S4 The specific structural features of the mitochondrial DNA of GTC.

Gene	From	To	Size/bp	aa	Start codon	stop codon	Gene spacer	Strand
tRNA-Phe	1	69	69					H
12S rRNA	70	1029	960				0	H
tRNA-Val	1030	1101	72				0	H
16S rRNA	1102	2793	1692				0	H
tRNA-Leu	2794	2869	76				0	H
NADH 1	2871	3845	975	325	ATG	TAA	1	H
tRNA-Ile	3850	3921	72				4	H
tRNA-Gln	3920	3990	71				-2	L
tRNA-Met	3992	4060	69				1	H
NADH 2	4061	5105	1045	348	ATG	T--	0	H
tRNA-Trp	5106	5176	71				0	H
tRNA-Ala	5178	5246	69				1	L
tRNA-Asn	5248	5320	73				1	L
tRNA-Cys	5353	5420	68				32	L
tRNA-Tyr	5422	5492	71				1	L
COXI	5494	7044	1551	517	GTG	TAA	1	H
tRNA-Ser	7045	7115	71				0	L
tRNA-Asp	7119	7192	74				3	H
COXII	7207	7897	691	230	ATG	T--	4	H
tRNA-Lys	7898	7973	76				0	H
ATPase 8	7975	8139	165	55	ATG	TAG	1	H
ATPase 6	8133	8815	683	227	ATG	TA-	-7	H
COXIII	8816	9600	785	261	ATG	TA-	0	H
tRNA-Gly	9601	9672	72				0	H
NADH 3	9673	10021	349	116	ATG	T--	0	H
tRNA-Arg	10022	10091	70				0	H
NADH 4L	10092	10388	297	99	ATG	TAA	0	H
NADH 4	10382	11763	1382	460	ATG	TA-	-7	H
tRNA-His	11764	11832	69				0	H
tRNA-Ser	11833	11901	69				0	H
tRNA-Leu	11903	11975	73				1	H
NADH 5	11976	13811	1836	612	ATG	TAA	0	H
NADH 6	13808	14329	522	174	ATG	TAG	-4	L
tRNA-Glu	14330	14398	69				0	L

Table S4 (continued)

Gene	From	To	Size/bp	aa	Start codon	stop codon	Gene spacer	Strand
Cytb	14403	15543	1141	380	ATG	T--	4	H
tRNA-Thr	15544	15615	72				0	H
tRNA-Pro	15615	15684	70				-1	L
D-loop	15685	16620	936				0	

Table S5 The specific structural features of the mitochondrial DNA of BG.

Gene	From	To	Size/bp	aa	Start codon	stop codon	Gene spacer	Strand
tRNA-Phe	1	69	69					H
12S rRNA	70	1031	962				0	H
tRNA-Val	1032	1103	72				0	H
16S rRNA	1104	2796	1693				0	H
tRNA-Leu	2797	2872	76				0	H
NADH 1	2874	3848	975	325	ATG	TAA	1	H
tRNA-Ile	3853	3924	72				4	H
tRNA-Gln	3923	3993	71				-2	L
tRNA-Met	3995	4063	69				1	H
NADH 2	4064	5108	1045	348	ATG	T--	0	H
tRNA-Trp	5109	5179	71				0	H
tRNA-Ala	5181	5249	69				1	L
tRNA-Asn	5251	5323	73				1	L
tRNA-Cys	5356	5423	68				32	L
tRNA-Tyr	5426	5496	71				2	L
COXI	5498	7048	1551	517	GTG	TAA	1	H
tRNA-Ser	7049	7119	71				0	L
tRNA-Asp	7122	7195	74				2	H
COXII	7209	7899	691	230	ATG	T--	13	H
tRNA-Lys	7900	7975	76				0	H
ATPase 8	7977	8141	165	55	ATG	TAA	1	H
ATPase 6	8135	8817	683	227	ATG	TA-	-7	H
COXIII	8818	9602	785	261	ATG	TA-	0	H
tRNA-Gly	9603	9674	72				0	H
NADH 3	9675	10023	349	116	ATG	T--	0	H
tRNA-Arg	10024	10093	70				0	H
NADH 4L	10094	10390	297	99	ATG	TAA	0	H
NADH 4	10384	11765	1382	460	ATG	TA-	-7	H
tRNA-His	11766	11834	69				0	H
tRNA-Ser	11835	11903	69				0	H
tRNA-Leu	11905	11977	73				1	H
NADH 5	11978	13813	1836	612	ATG	TAA	0	H
NADH 6	13810	14331	522	174	ATG	TAA	-4	L
tRNA-Glu	14332	14400	69				0	L

Table S5 (continued)

Gene	From	To	Size/bp	aa	Start codon	stop codon	Gene spacer	Strand
Cytb	14405	15541	1137	379	ATG	TAA	4	H
tRNA-Thr	15546	15617	72				4	H
tRNA-Pro	15617	15686	70				-1	L
D-loop	15687	16623	937				0	

Table S6 The proportions and counts of bases in GTC and BG mitochondrial DNA.

Fish	Base proportions (%)						Base Counts					
	A	C	G	T	A+T	G+C	A	C	G	T	A+T	G+C
GTC	31.45	27.56	15.99	25.00	56.45	43.55	5,227	4,580	2,658	4,155	9,382	7,238
BG	31.23	27.91	16.19	24.67	55.90	44.10	5,192	4,639	2,691	4,101	9,293	7,330

Table S7 The survival rates throughout the 180-day fattening period.

Fish Type	Initial Number (individuals)	Final Number (individuals)	Average Survival Rate (%)
BSB	300	83	27.67±1.70
GTC	300	100	33.33±3.30
BG	300	95	31.67±2.87

Note: Data are presented as mean ± standard deviation (SD) from each group (n = 20).

Table S8 Fatty acid content of BSB, GTC, and BG.

Fatty acids	BSB	GTC	BG
C14:0 (g/100g)	0.0063±0.0012	0.0052±0.0003	—
C16:0 (g/100g)	0.1373±0.0163	0.1195±0.0119	0.1096±0.0407
C16:1 (g/100g)	0.0245±0.0062	0.0165±0.0018	0.0064±0.0044 *#
C18:0 (g/100g)	0.0774±0.0323	0.0753±0.0344	0.0592±0.0276
C18:1n9c (g/100g)	0.2800±0.0171 #	0.1703±0.0412 *	0.0899±0.0497 *
C18:2n6c (g/100g)	0.0885±0.0308	0.0676±0.0475	0.0502±0.0047
C20:1 (g/100g)	0.0105±0.0010 #	0.0064±0.0012 *	0.0043±0.0010 **
C20:2 (g/100g)	0.0053±0.0005 #	0.0039±0.0001 *	0.0045±0.0001 ##
C20:3n6 (g/100g)	0.0128±0.0026	—	0.0107±0.0020
C22:1n9 (g/100g)	0.0089±0.0083	0.0115±0.0079	—
C20:4n6 (g/100g)	0.0312±0.0220	—	0.0247±0.0183
C20:5n3 (g/100g)	0.0059±0.0007	0.0084±0.0038	0.0068±0.0001
C24:1 (g/100g)	—	0.0044±0.0003	0.0039±0.0006
C22:6n3 (g/100g)	0.0342±0.0098	0.0294±0.0086	0.0378±0.0110
∑SFAs	0.2210±0.0395	0.2000±0.0405	0.1688±0.0681
∑MUFAs	0.3239±0.0304 #	0.2090±0.0392 *	0.1045±0.0544 **
∑PUFAs	0.1779±0.0645	0.1092±0.0579	0.1346±0.0349
∑UFAs	0.5018±0.0465	0.3182±0.0971	0.2391±0.0406 **

"∑ SFAs" represents saturated fatty acids; "∑ UFAs" represents unsaturated fatty acids; "∑ MUFAs" represents monounsaturated fatty acids; "∑ PUFAs" represents polyunsaturated fatty acids. "*" indicates a significant difference with BSB, * $p < 0.05$, ** $p < 0.01$; "#" indicates a significant difference with GTC, # $p < 0.05$, ## $p < 0.01$. Note: Date are presented as the means ± SD, n = 9 replicates per group.

Table S9 Amino acid content (mg/g N), AAS, CS and EAAI of BSB, GTC, and BG.

Index	Essential amino acids	BSB	GTC	BG	FAO/WHO standard	Egg protein standard
Amino acid content	Thr	230.16	232.29	233.19	250	292
	Val	264.10	268.34	265.46	310	411
	Met + Cys	146.04	153.22	153.37	220	386
	IIe	236.15	241.62	234.12	250	331
	Leu	438.19	443.74	437.56	440	534
	Phe + Tyr	406.71	412.23	401.84	380	565
	Lys	528.04	537.71	528.18	340	441
AAS	Thr	0.92	0.93	0.93		
	Val	0.85 [2]	0.87 [2]	0.86 [2]		
	Met + Cys	0.66 [1]	0.70 [1]	0.70 [1]		
	IIe	0.94	0.97	0.94		
	Leu	1.00	1.01	0.99		
	Phe + Tyr	1.07	1.08	1.06		
	Lys	1.55	1.58	1.55		
CS	Thr	0.79	0.80	0.80		
	Val	0.64 [2]	0.65 [2]	0.65 [2]		
	Met + Cys	0.38 [1]	0.40 [1]	0.40 [1]		
	IIe	0.71	0.73	0.71		
	Leu	0.82	0.83	0.82		
	Phe + Tyr	0.72	0.73	0.71		
	Lys	1.20	1.22	1.20		
EAAI		71.62	73.17	72.29		

"1" is the first limiting amino acid; "2" is the second limiting amino acid.

Supplementary Explanation

1. Supplementary notes for Figure 5.

Figure 5: The nucleotide sequence alignment of 5S rDNA fragments in BSB, GTC, and BG. (A) Nucleotide sequence alignment of 5S rDNA fragments in BSB (188 bp), GTC (188 bp), and BG (188 bp). (B) Nucleotide sequence alignment of 5S rDNA fragments in BSB (376 bp), GTC (387 bp), and BG (376 bp). 5S rRNA is underlined. The blue boxes indicate the regulatory sequences, A-box, intermediate elements, C-box, and TATA-box. The red boxes indicate the PCR primers. The green boxes indicate species-specific regions for BSB and BG. The blue boxes indicate species-specific regions for GTC and BG.

2. Supplementary notes for Figure 6.

Figure 6: The nucleotide sequence alignment of *Sox9* gene fragments in BSB, GTC, and BG. Nucleotide sequence alignment of *Sox9* gene fragments in BSB (714 bp), GTC (718 bp), BG-I (714 bp), and BG-II (718 bp). The dark purple shadow boxes indicate the bases that are identical in BSB, GTC, and BG. The red boxes indicate the bases with identical sequences in BG and GTC.

3. Supplementary analysis of the *Sox* genes.

Sequence analysis revealed that the 200 bp fragment from all three species corresponded to a 215 bp sequence. Sequence alignment in NCBI showed that the 215 bp fragment from BSB and BG contained both *Sox*1 and *Sox*11 genes, while the 215 bp fragment from GTC only contained the *Sox1* gene. Moreover, the 700 bp fragments from BSB and GTC were identified as 714 bp and 718 bp, respectively, while the hybrid offspring BG exhibited both 714 bp and 718 bp fragments. All 700 bp fragments from the three species were identified as belonging to the *Sox*9 gene. The *Sox9* gene nucleotide sequence similarity analysis revealed that the 714 bp fragment from BG-I showed 99.86% similarity to BSB, while the 718 bp fragment from BG-II showed 99.72% similarity to GTC.

4. Supplementary of water quality logs and food composition|ratios.

Throughout the entire culture period, the experimental fish were maintained under a controlled photoperiod of 12 h light : 12 h dark, and fed a commercial compound diet with a fixed ratio of 6:1 (sinking feed to floating feed), twice daily at 09:00 and 17:00. The culture system was supplied with a continuous flow (24 h/day) of water, maintained at 22 ~ 25℃, which ensured adequate water exchange and sustained dissolved oxygen levels above 6.5 mg/L.

5. Supplementary of the formula for calculating protein nutritional index.

The amino acid content relative to nitrogen was calculated as:

AA (mg/g N) = (AA% / Protein%) × 6.25 × 1000

The Amino Acid Score (AAS) and Chemical Score (CS) were determined by comparing the test protein's amino acid profile to the FAO/WHO reference pattern and whole egg protein, respectively:

AAS = [AA (mg/g) in test protein] / [AA (mg/g) in FAO/WHO pattern]

CS = [AA (mg/g) in test protein] / [AA (mg/g) in whole egg protein]

The Essential Amino Acid Index (EAAI) was calculated as the geometric mean of the chemical scores for all essential amino acids:

$EAAI = 100 \times \sqrt[n]{(CS_1 \times CS_2 \times CS_3 \times \ldots \times CS_n)}$

where:

AA is the content of an individual amino acid;

Protein% is the total protein content (g/100 g);

6.25 is the conversion factor (from N to protein);

CS_1, CS_2, ..., CS_n are the chemical scores for each of the “n” essential amino acids included in the evaluation.

6. Supplementary of detailed information for the two standards: GB 5009.5-2016 and GB 5009.6-2016.

GB 5009.5-2016: GB 5009.5-2016; National Food Safety Standard-Determination of Protein in Foods. National Standards of the People's Republic of China: Beijing, China, 2016.

GB 5009.6-2016: GB 5009.6-2016; National Food Safety Standard-Determination of Fat in Foods. National Standards of the People's Republic of China: Beijing, China, 2016.
